# Sialadenoma papilliferum: clinicopathologic, Immunohistochemical, molecular analyses of new five cases and review of the literature

**DOI:** 10.1186/s13000-021-01084-5

**Published:** 2021-03-12

**Authors:** Shuai Chen, Jie Peng, Chuantao Yuan, Lin Sun, Renya Zhang, Yan Sun

**Affiliations:** 1 Department of Pathology; Key Laboratory of Cancer Prevention and Therapy; Tianjin’s Clinical Research Center for Cancer; National Clinical Research Center for Cancer, Tianjin Cancer Institute and Hospital, Tianjin Medical University, Tianjin, PR China; 2 Department of Pathology, Affiliated Hospital of Jining Medical University, Jining, Shandong Province PR China; 3 Department of Pathology, ZiBo Central Hospital, Zibo, Shandong Province PR China

**Keywords:** Sialadenoma papilliferum, Clinical features, Histopathology, Immunohistochemistry, BRAF

## Abstract

**Background:**

Sialadenoma papilliferum (SP) is an extremely rare benign neoplasm of salivary glands. To explore and define the clinicopathological features of SP, we retrospectively analyzed 89 cases previously reported and five new cases.

**Methods:**

The clinical features, histopathology, immunohistochemistry and molecular analysis of our cases were further performed and the related literatures were reviewed and analyzed.

**Results:**

Combining 89 cases from the literature with our cases, the hard palate was the most common locations for SP. However, two of our cases were rarely located in the esophageal mucosa. Among all cases, the male gender was more affected, with the average age and median age of 61.8 and 62 years, respectively. Conventional histomorphologically, SP was characterized by complex papillary structures with a biphasic growth pattern of exophytic squamous component and endophytic glandular component. The glandular structures were lined by a double layer of epithelium composed of flattened or cuboidal basal cells and a cuboidal or columnar luminal cells formed papillary infoldings into the ductal lumina. Immunohistochemically, the luminal epithelial configurations showed strong expression of CK7 along the luminal cell membrane, while the basal myoepithelia displayed strong nuclear p63 expression. In both the glandular and squamous tumour components showed BRAF V600E-positive immunostaining and *BRAF* V600E mutation.

**Conclusion:**

For the first time, we have comprehensively aggregated and analyzed 90 cases sialadenoma papilliferum from almost all previous publications, and further explored the clinicopathological features of SP; concordantly, this study demonstrated that SP shows a papillomatous growth pattern with exophytic and endophytic proliferation of ductal epithelium composed of double-layered cells harboring *BRAF* V600E mutation. Additionly, adequate treatment for SP is surgical excision, with a favorable prognosis in patients.

## Background

Sialadenoma papilliferum (SP) is a rare benign neoplasm [1–3], estimated to account for less than 1% of all minor salivary gland tumours and 3–5% of head and neck tumours [2–7]. It was described initially in 1969 by Abrams and Finck, because of its histomorphology closely resembling that of the syringocystadenoma papilliferum of cutaneous adnexal origin, and a total of 90 cases were reported by 2021 [2–5, 8]. SP is characterized by coexisting papillar and glandular configurations, which occurs mainly in the palate, especially the hard palate. It can also occur in the soft palate, buccal mucosa, nasal cavity, upper lip, parotid glands, and rarely in the bronchus and esophagus [9, 10]. SP usually presents as a painless exophytic papillary mass with the peak incidence in the fifth, sixth and seventh decades of life [2, 5, 6, 11–16]. The prognosis of SP is mostly good; and in single cases may have recurrence [10] or malignant transformation [12, 13]. Given the rareness of SP and difficulty in distinguishing it from other malignant tumours, the experience gained from the present cases and thorough analysis of medical literature may be useful for pathologists and clinicians on the correct diagnosis of this disease.

## Materials and methods

### Patients and samples

With Institutional Board Review approval, the Department of Pathology of the Affiliated Hospital of Jining Medical University and consultation archives from 2017 to 2019 were searched for cases of SP and analyzed for their clinicopathological features. In addition, all available hematoxylin and eosin-stained sections were reviewed and confirmed by two pathologists with expertise in head and neck tumour pathology. Apart from this, a thorough English language literature search was performed primarily through Google Scholar, PubMed, and the different editions of WHO classifications of salivary gland tumours using the keyword sialadenoma papilliferum, which included single case reports and short series of SP since 1969. Consequently, a total of 48 articles were remained for the literature analysis, and the following indexes were taken into account: patient gender, age at diagnosis, lesion occurrence site, lesion size, progression time, clinical diagnosis, clinical features, follow-up period, and recurrence. Also excluded from our study were cases with questionable histopathologic features or without available microscopic images.

### Immunohistochemistry

The immunohistochemical analysis was performed on paraffin-embedded sections using the EnVision two-step method. Primary antibodies used in the study were displayed as follows: CK7 (MAB-0828, MX053), CK5/6 (MAB-0744, MX040), p63 (MAB-0694, MX013), CK8 (MAB-0670, MX004), S-100 (Kit-0007, 4C4.9), and Ki-67 (MAB-0672, MX006), Ready-to-use, Maixin Bio, Fujian, China. Anti-BRAF V600E (VE1, Ventana) antibody was performed on Ventana BenchMark GX autostainer (Ventana) followed by the Optiview DAB Detection Kit (Ventana). Appropriate positive and negative controls were performed concomitant for all the markers tested.

### Molecular analyses

The unstained paraffin-embedded sections were collected for DNA extraction (QIAamp DNA FFPE Tissue Kit, Qiagen, Hilden, Germany). And then DNA was amplified by polymerase chain reaction using primers for exon 15 of BRAF (HotStarTaq Master Mix kit (Qiagen), 5′-TCA TAA TGC TTG CTC TGA TAGGA-3′ (BRAF-Exon15-F), 5′-GGC CAA AAA TTT AAT CAG TGGA-3′(BRAF-Exon15-R)). The amplified products were purified using a QIAquick Spin Kit (Qiagen), and then purified products were sequenced with a BigDye Terminator v3.1 Cycle Sequencing Kit (Applied Bio-systems, Foster City, CA, USA) on an ABI Prism 3700 instrument (Applied Biosystems). The confirmed assay was repeated for mutational specimens.

## Results

### Clinical features

There were five cases of SP from our hospital, including three males and two females, and age at diagnosis ranged from 50 to 78 years with an average age of 62 years. Four patients were found by accident in physical examination, only with slight local numbness but no obvious pain. In addition, one patient was treated for palatal tumours because of pain for 1 month. Two of the five cases occurred in the palate, one in nasal cavity and two in esophagus. All patients underwent tumour resection and were sent to the pathology department for pathological examination, with size of the mass ranging from 0.5 to 1.0 cm. Patients were followed for 6 to 22 months, and their prognosis was good without recurrence and worsening progression.

**Fig. 1:**
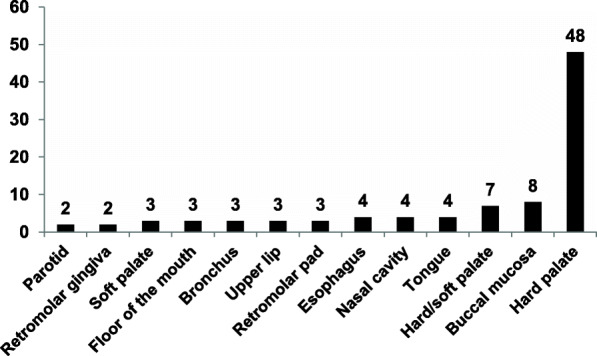
Anatomic location of sialadenoma papilliferum (94 cases)

**Fig. 2:**
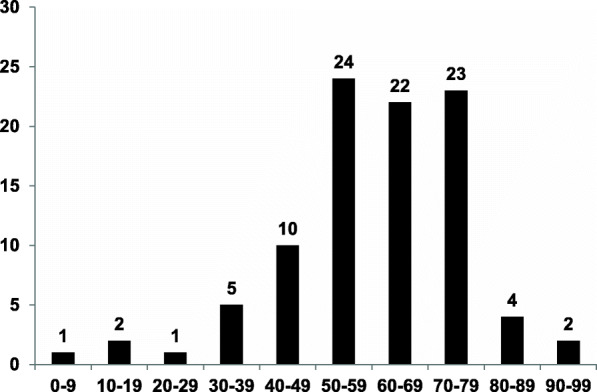
Age distribution of sialadenoma papilliferum (94 cases)

Combining 94 cases from the literature with our cases, the hard palate was the most common location, accounting for 60%, and others included the junction of the hard and soft palate, soft palate, buccal mucosa, nasal cavity, upper lip, parotid glands, etc. (Fig. 1 and Table 1). Moreover, two of our cases were rarely located in the esophageal mucosa. There were 56 men and 38 women, showing preference for males, and age at diagnosis ranged from 2 to 96 years, with mean age and median age were 61.8 and 62 years, respectively (Fig. 2). Duration of the lesions ranged from 1 month to over 8 years, with 49.8 months of average duration. The misinterpreted clinical diagnosis included squamous papilloma, fibroma, mucocele, salivary gland neoplasm, warty dyskeratoma, cystadenoma, cystadenocarcinoma or verrucous carcinoma.

**Table 1:** Clinical features of sialadenoma papilliferum (95 cases, but 94 cases available)

Reference	Age	Sex	Location	Duration	Size (cm)	Clinical diagnosis	Clinical features	Follow-up
Case 1	78	M	Hard palate	1 mo	0.5	Squamous papilloma	Yellow-brown mucosa	NED-19 mo
Case 2	56	F	Hard palate	5 mo	1.0	Squamous papilloma	Hard and clear tubercle	NED-15 mo
Case 3	72	M	Nasal cavity	2 mo	1.0	Squamous papilloma	Hard and clear tubercle	NED-9 mo
Case 4	50	F	Esophagus	9 mo	0.6	Squamous papilloma	Pink papillary pedunculated	NED-22 mo
Case 5	53	M	Esophagus	1 mo	0.8	Squamous papilloma	Pink papillary pedunculated	NED-6 mo
Bobos et al. [1]	53	M	Bronchus	4 mo	2.2	N/A	Exophytic papillary yellowish tumour	N/A
Campisi et al. [2]	66	M	Bronchus	N/A	1.5	Pulmonary nodule	Micro-cystic neoplasm	NED-36 mo
Abrams et al. [4]	71	M	Parotid	10–12 yrs	7.5	Low-grade malignancy	Fungating cauliflower-like mass	NED-18 mo
Abrams et al. [4]	57	M	Hard/soft palate	3 mo	1.5	N/A	Verrucous lesion	NED-19 mo
Hsieh et al. [5]	65	M	Hard palate	N/A	0.3	N/A	N/A	N/A
Hsieh et al. [5]	77	M	Hard palate	N/A	0.2	N/A	N/A	N/A
Hsieh et al. [5]	83	F	Hard palate	N/A	0.3	N/A	N/A	N/A
Hsieh et al. [5]	52	M	Hard palate	N/A	0.5	N/A	N/A	N/A
Hsieh et al. [5]	2	M	Buccal mucosa	N/A	0.4	N/A	N/A	N/A
Hsieh et al. [5]	91	F	Hard palate	N/A	0.5	N/A	N/A	N/A
Hsieh et al. [5]	58	F	Tongue	N/A	0.6	N/A	N/A	N/A
Hsieh et al. [5]	77	F	Hard palate	N/A	0.2	N/A	N/A	N/A
Hsieh et al. [5]	36	F	Hard palate	N/A	0.2	N/A	N/A	N/A
Hsieh et al. [5]	61	M	Buccal mucosa	N/A	0.2	N/A	N/A	N/A
Hsieh et al. [5]	73	F	Hard palate	N/A	0.3	N/A	N/A	N/A
Hsieh et al. [5]	77	F	Hard palate	N/A	0.4	N/A	N/A	N/A
Hsieh et al. [5]	64	M	Hard palate	N/A	0.3	N/A	N/A	N/A
Fowler et al. [6]	55	F	Hard palate	Several mo	0.3	Papilloma	Exophytic pebbly pink with stalk	N/A
Fowler et al. [6]	50	M	Hard palate	> 15 yrs.	0.8	Papilloma,fibroma, hemangioma	Exophytic pink/red	N/A
Fowler et al. [6]	62	M	Hard palate	N/A	N/A	Papilloma	Papilloma-appearing lesion	N/A
Fowler et al. [6]	63	M	Hard palate	N/A	N/A	Mucocele, fibroma	Raised mass	N/A
Fowler et al. [6]	57	M	Hard palate	1 mo	0.4	Papilloma	Red with stalk, fingerlike	N/A
Fowler et al. [6]	48	F	Hard palate	N/A	0.5	Fibroma, salivary gland tumour	Red,slightly elevated	N/A
Fowler et al. [6]	76	F	Hard palate	2.5 mo	1.3	Carcinoma	White rough lesion	N/A
Gera et al. [7]	96	F	Nasal cavity	30 yrs	0.8	N/A	Yellow-brown mucosa	NED-9 mo
Loehn et al. [8]	65	M	Parotid	8 yrs	7.5	Sialadenoma papilliferum	Pink, exophytic, fungating tumour	NED-14 mo
Oze et al. [9]	67	F	Nasal cavity	2 yrs	0.8	Sialadenoma papilliferum	Recurrent epistaxis	NED-4 mo
Pimentel et al. [10]	67	F	Buccal mucosa	12 mo	2	N/A	Sessile mass; recurrence	Rec-36 mo
Reis de et al. [11]	20	M	Upper lip	N/A	1.6	Mucocele	Nodular mass	NED-21 mo
Ponniah et al. [12]	30	M	Floor of the mouth	N/A	1.5	Sialadenoma papilliferum	An asymptomatic, exophytic, slightly papillary lesion	NED-8 mo
Shimoda et al. [13]	79	F	Hard/soft palate	N/A	4	Sialadenoma papilliferum	Exophytic pink-white papillary mass	NED-1 mo
Mahajan et al. [14]	18	M	Upper lip	12 yrs.	0.8	Infected hemangioma	Firm tumour	NED
Kubota et al. [15]	62	M	Hard palate	3 mo	1	N/A	White, exophytic	NED-13 mo
Atarbashi-Mogha dam et al. [16]	50	F	Hard palate	12 mo	1	N/A	Reddish mass with slightly papillary	NED-48 mo
Gomes et al. [17]	53	M	Hard palate	3 yrs.	1	Papilloma, vascular	Pedunculated red papillary mass	NED
Gomes et al. [17]	52	F	Soft palate	4 yrs	0.5	Fibrous polyp	Firm pedunculated mass	NED
Ubaidat et al. [18]	72	M	Hard palate	N/A	0.4	N/A	Exophytic growth (0.6 cm on gross)	NED-3 mo
Ubaidat et al. [18]	58	M	Hard palate	N/A	0.5	Melanoma	Ulcerated and pigmented	NED
Brannon et al. [19]	69	F	Hard palate	N/A	N/A	Squamous papilloma	Slow growing exophytic mass	N/A
Brannon et al. [19]	53	F	Hard palate	3 mo	N/A	Squamous papilloma	Slow growing exophytic mass	N/A
Brannon et al. [19]	31	F	Hard palate	4 yrs.	N/A	Squamous papilloma	Slow growing exophytic mass	N/A
Argyres et al. [20]	50	M	Hard palate	Several mo	0.5	Squamous cell car-cinoma	Irm exophytic mass	N/A
Markopoulos et al. [21]	50	M	Hard palate	12 yrs	0.5	N/A	Papillary mass	N/A
Asahina et al. [22]	50	M	Hard palate	6 mo	0.5	Fibrous polyp	Cauliflower like mass, white/pink	NED-24 mo
Maiorano et al. [23]	56	M	Hard palate	N/A	0.5	Squamous papilloma	N/A	NED-18 mo
Maiorano et al. [23]	37	F	Hard palate	N/A	1	Verrucous leukoplakia	N/A	NED-48 mo
Maiorano et al. [23]	60	M	Buccal mucosa	N/A	0.8	Squamous papilloma	N/A	NED-96 mo
Maiorano et al. [23]	46	M	Hard palate	N/A	1.4	Salivary gland tumour	N/A	NED-36 mo
Maiorano et al. [23]	50	M	Hard palate	N/A	1.8	Salivary adenoma	N/A	NED-6 mo
Van der Wal et al. [24]	46	M	Hard/Soft palate	10 yrs	0.5	Fibroepithelial polyp	Pedunculated firm exophytic mass	NED-12 mo
Nakahata et al. [25]	N/A		N/A	N/A	N/A	N/A	N/A	N/A
Papanicolaou et.al [26].	46	M	Hard palate	N/A	0.5	N/A	Red firm exophytic growth	N/A
Fantasia et al. [27]	87	F	Hard palate	Several mo	N/A	Irritated papilloma	Exophytic red papillary lesion	N/A
Fantasia et al. [27]	77	M	Buccal mucosa	N/A	N/A	N/A	N/A	N/A
Fantasia et al. [27]	48	F	Hard palate	N/A	N/A	N/A	N/A	N/A
Fantasia et al. [27]	45	M	Hard palate	N/A	N/A	N/A	N/A	N/A
Fantasia et al. [27]	60	F	Upper lip	N/A	N/A	N/A	N/A	N/A
Mitre [28]	42	F	Hard/soft palate	7 mo	0.4	N/A	Red warty lump	N/A
Rennie et al. [29]	77	M	Hard/soft palate	N/A	1	Squamous papilloma	Firm warty papule	Rec-36 mo
Miyamoto et al. [30]	53	M	Buccal mucosa	5 mo	0.8	N/A	Painless, exophytic mass	NED-24 mo
Puts et al. [31]	78	M	Hard palate	N/A	1.6	N/A	Exophytic papillary growth	NED
Sunil et al. [32]	58	F	Hard palate	1 mo	1	Papilloma/fibroma	Exophytic erythematous	N/A
Shirasuna et al. [33]	56	F	Hard palate	N/A	0.7	Squamous papilloma	Pedunculated papillary mass	NED-20 mo
Wertheimer et al. [34]	32	M	Hard palate	N/A	0.5	Papilloma	Dome-shaped mass	NED-18 mo
Wertheimer et al. [34]	43	M	Soft palate	8 yrs	0.5	N/A	Papillary mass, recently ulcerated	NED-30 mo
Nasu et al. [35]	62	F	Hard palate	6 mo	0.6	N/A	Papillary exophytic mass, slow growth	N/A
McCoy et al. [36]	77	F	Buccal mucosa	N/A	0.7	N/A	Papillary growth, indurated	NED
Drummond et al. [37]	71	M	Retromolar pad	N/A	0.5	N/A	Pink papillary, pedunculated	NED-6 mo
Jensen et al. [38]	48	M	Hard palate	10 yrs	0.8	Squamous papilloma	Pedunculated papillary lesion	N/A
Su et al. [39]	70	F	Esophagus	20 mo	1.0	Adenocarcinoma	Broad-based polypoid tumour	NED-12 mo
Rouse et al. [40]	81	M	Esophagus	36 mo	1.5	Esophageal adenoma	Pedunculated polyp	NED-12 mo
Honda et al. [41]	75	M	Bronchus	2 mo	0.5	N/A	Exophytic papillary lesion	NED-8 mo
Freedman et al. [42]	68	M	Hard palate	N/A	0.3	N/A	Raised sessile papillary lesion	NED-21 mo
Freedman et al. [42]	68	M	Hard palate	1 mo	0.5	N/A	Raised sessile papillary lesion	NED-19 mo
Anuradha et al. [43]	65	M	Floor of the mouth	12 mo	0.8	Papilloma	Soreness in mouth	NED-12 mo
Nakaguro et al. [44]	71	F	Soft palate	N/A	0.4	N/A	N/A	N/A
Nakaguro et al. [44]	80	F	Retromolar gingiva	N/A	0.8	N/A	N/A	N/A
Nakaguro et al. [44]	45	F	Hard palate	N/A	0.7	N/A	N/A	N/A
Nakaguro et al. [44]	75	M	Buccal mucosa	N/A	0.6	N/A	N/A	N/A
Nakaguro et al. [44]	67	M	Retromolar gingiva	N/A	2.3	N/A	N/A	N/A
Nakaguro et al. [44]	79	M	Hard palate	N/A	0.6	N/A	N/A	N/A
Nakaguro et al. [44]	75	M	Tongue	N/A	0.6	N/A	N/A	N/A
Nakaguro et al. [44]	63	F	Tongue	N/A	0.9	N/A	N/A	N/A
Nakaguro et al. [44]	78	F	Floor of the mouth	N/A	1.3	N/A	N/A	N/A
Nakaguro et al. [44]	66	F	Tongue	N/A	0.8	N/A	N/A	N/A
Hamilton et al. [45]	15	F	Nasal cavity	N/A	N/A	Salivary neoplasm	Chronic nasal obstruction	NED-12 mo
Koc et al. [46]	72	M	Retromolar pad	12 mo	3	N/A	Mass with a papillamatous hemorrhagic surface	NED-12 mo
Ide et al. [47]	67	M	Retromolar pad	12 mo	3	N/A	Cauliflflower-like mass	N/A

*mo* Month(s), *yrs* Years, *N/A* Not available, *NED* No evidence of disease

### Macroscopy

The tumour varied in sizes, from 0.2 to 3.0 cm in 70 cases known, with an average size of 0.81 cm, excluding two cases from the parotid gland (7.5 cm) and another two cases from the palate and left lower gingiva (4.0 cm). Gross observation showed that the cut surface of the lesion was slightly solid with grey-white to grey red colour, clear margins, and the papillary surface.

### Histopathology features

**Fig. 3:**
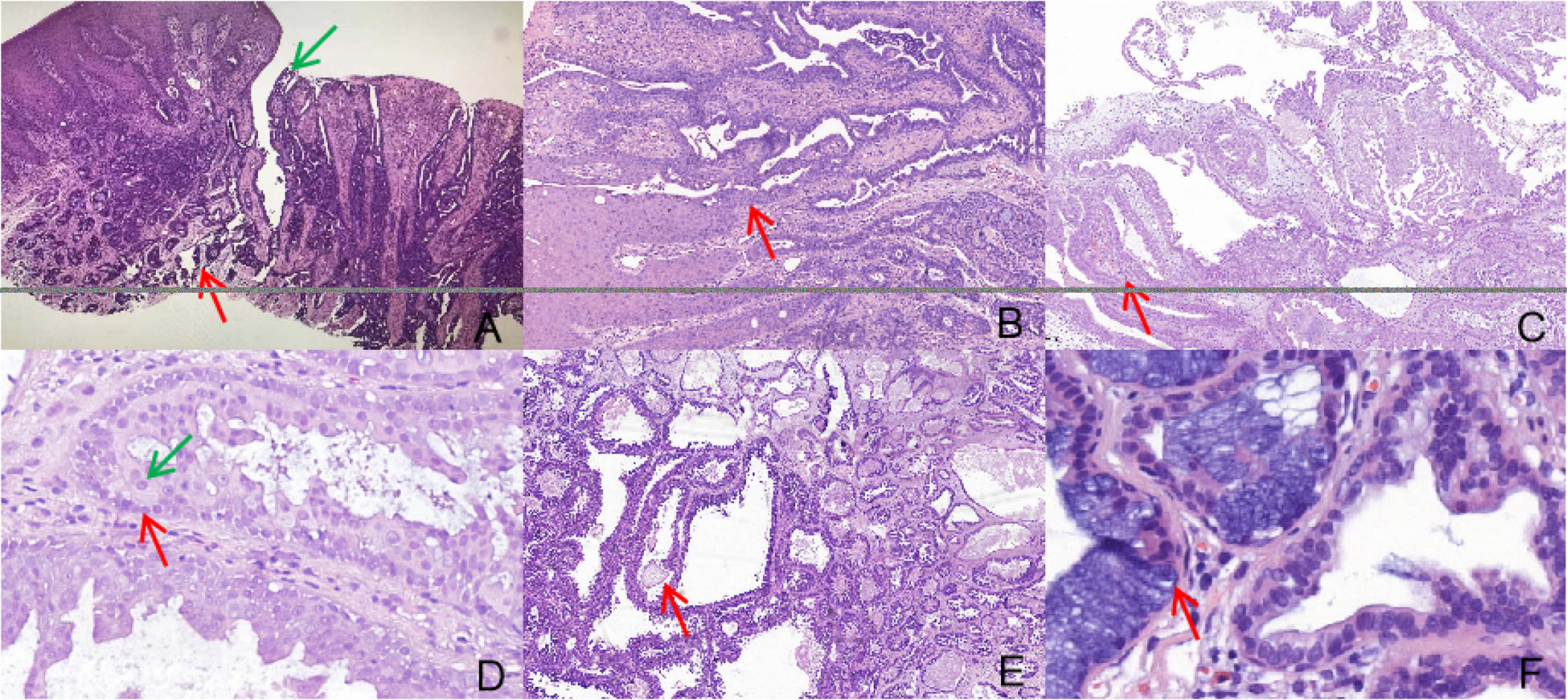
Pathological manifestations of Sialadenoma papilliferum. **a** SP shows a biphasic growth pattern with an exophytic papillary component surfaced by a keratotic squamous epithelium (Green arrow) and an endophytic adenomatous component of the underlying lesion (Red arrow) (20x). **b** Papillary frond surfaced by stratified squamous epithelium is contiguous with columnar ductal epithelium (Red arrow) (100x). **c** The surface papillary structure of the lumen supported by thin fibrovascular core often protrudes into the lumen (Red arrow) (50x). **d** The ductal lumen is double-layered, lined by luminal cuboidal to columnar cells (Green arrow) and cuboidal to flattened basal cells (Red arrow) (400x). **e** Localized enlargement of the cyst and eosinophilic deposition in the lumen (Red arrow) (50x). **f** Mucinous cells in tumour area (Red arrow) (400x)

Histologically, the tumours in our cases were characterized by biphasic differentiation, consisting of exophytic papillary structures covered by stratified squamous epithelium and endophytic glandular structures below the mucosa (Fig. 3a). The surface of papillary structures was covered with multiple layers of squamous epithelium, and the squamous epithelium was locally contiguous with a proliferation of papillomatous ductal epithelium located underneath the mucosal surface and extending downward into the deeper stroma (Fig. 3b). These papillas supported by fibrovascular connective tissue core often protruded into the lumen (Fig. 3c). The ductal epithelium was double-layered or multilayered structures, lined by luminal cuboidal to columnar cells and cuboidal to flattened basal cells (Fig. 3d). The luminal cells had round to oval, bland nuclei and inconspicuous nucleoli. The nuclei of regional tumours were enlarged with clear nucleoli, but without atypia. Inflammatory cell infiltration, including plasma cells, lymphocytes and neutrophils, was seen around the lumen and the connective tissue. Enlarged cysts with eosinophilic deposits (Fig. 3e) and accidental areas of oncocytic metaplasia can be seen in the ductal structures of 3 cases, and mucinous cells and necrosis were seen locally in 2 cases. In addition, The tumour area showed normal cell morphology and mucinous cells can be seen in some tumour areas (Fig. 3f).

### Immunohistochemical features

**Fig. 4:**
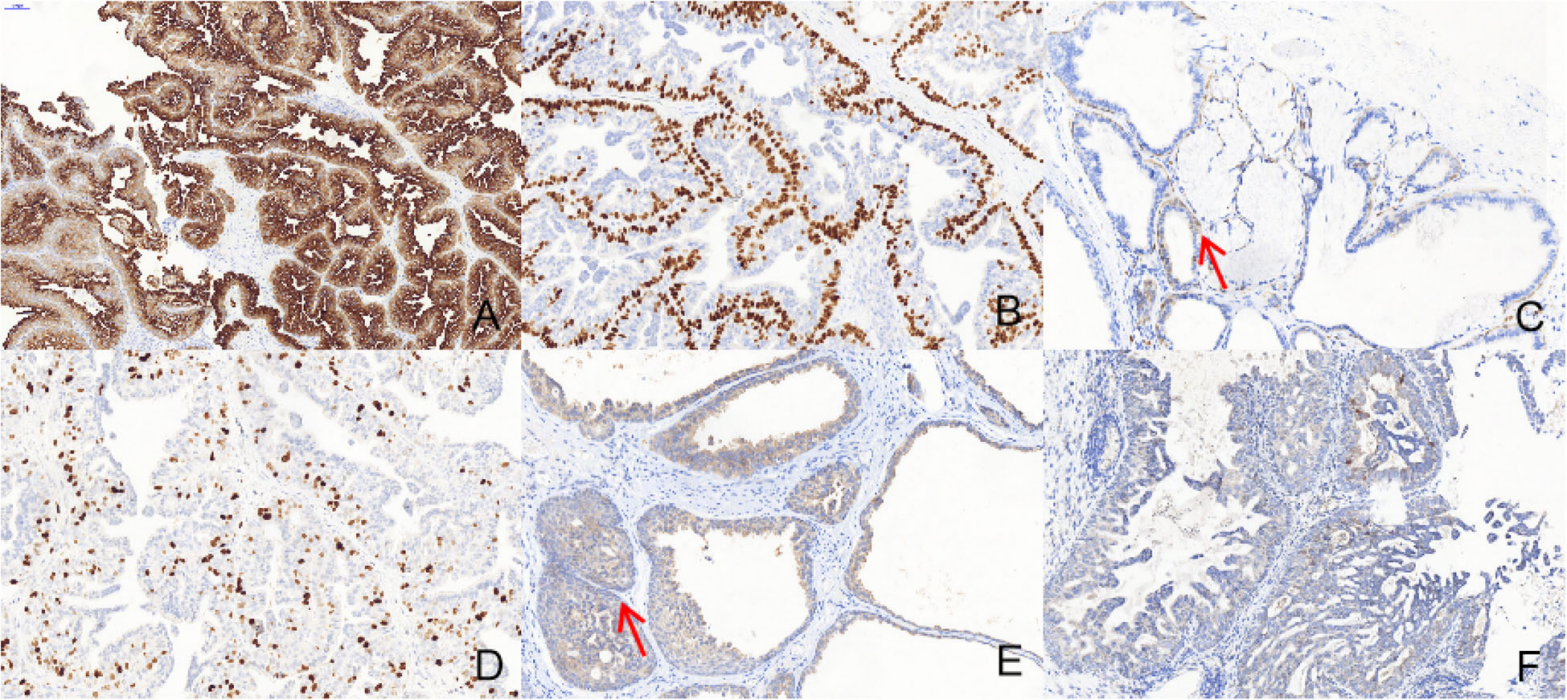
Immunohistochemical performance of Pathological manifestations. **a** CK7 is strongly positive in the luminal cells (100x). **b** p63 shows strong positive staining in the basal cells (100x). **c** S-100 shows positive staining in the basal cells (Red arrow) (100x). **d** Ki67 ranges from 20 to 30% (100x). **e** The expression of BRAF V600E is observed in both the glandular and squamous components (Red arrow) (100x). **f** BRAF V600E immunohistochemistry in the third case shows a weak staining (100x)

Immunohistochemical studies of our cases have shown that CK7 (Fig. 4a) and CK8 were strongly expressed in the ductal luminal cells, while p63 (Fig. 4b), CK5/6 and S-100 (Fig. 4c) were strongly expressed in the basal cell layer but were negative in the luminal cells. Ki67 decorated less than 30% lesional cells in all cases (Fig. 4d and Table 2). BRAF V600E in both the glandular and squamous tumour components showed a moderate or weak staining (Fig. 4e, f).

**Table 2:** Immunohistochemical findings of sialadenoma papilliferum (our five cases)

	Luminal cells	Basal cells
BRAF V600E	++	–
CK7	++	–
CK8	++	–
CK5/6	–	++
p63	–	++
S-100	–	+
Ki-67	5%	20–30%

*(+)* focal staining, *(++)* diffuse staining

### Molecular features

**Fig. 5:**
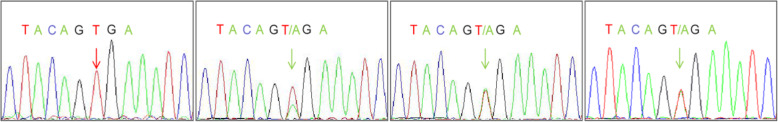
Molecular features of sialadenoma papilliferum. *BRAF* wild type (Red arrow); *BRAF* p.V600E c.1799 T > A (Green arrow). *BRAF* mutation is confirmed in three of four cases by Sanger sequencing

Molecular analysis revealed that *BRAF* mutation was confirmed in three of four cases, because of one case lacking sufficient tissue for testing (Fig. 5).

### Treatment and follow up

Our patients were followed for 6 to 22 months, and their prognosis was good without recurrence and worsening. According to the literature review, conservative surgery was documented in 56 cases and treatment was not specified in 6 cases. And one case from the left lower gingiva with malignant transformation invading the mandible was treated by partial mandibular resection and cervical lymph node dissection. Another case from the parotid gland, considering the preservation of the facial nerve, only superficial parotidectomy was performed. In addition, the follow-up information of 37 cases was available, in which the length of follow-up period of the patients ranged from 1 month to 96 months, with an average length of 31 months. Three cases with malignant transformation of SP have been recognized, and only two recurrences were recorded, each at 36 months after initial treatment, which indicated a recurrence rate of 6.5%.

## Discussion

SP is a subtype of intraductal papilloma, a rare benign tumour commonly found in older adults with hard palate, accounting for approximately 80%. It can also occur in the parotid gland, submandibular gland, nasopharynx and esophagus [2, 3, 17–31]. Clinically, there are generally no obvious clinical symptoms with mostly painless growth, but sometime papillary erythema or pedicled lumps, occasional ulcers [3, 32–43]. Grossly, most of the lesions presents as a round to oval mass with white-colored and papillary surface, sharing about 80%.

Histologically, SP is formed by mucosal surface epithelium and ductal epithelium of the salivary gland which proliferate outward and inward simultaneously, with the characteristics of biphasic proliferation of squamous and ductal epithelium [11, 44]. Generally, SP has two components [5, 6]: (1) superficial papillary structure: stratified squamous epithelium covered with incomplete keratinization, in addition to acanthosis or acanthosis cell edema; (2) ductal structure: the lumen-like structure lined by two or more layers of columnar and cuboidal cells is formed under the mucosa, and the ductal lumina can be mesh-like, fissure-like or expanded into a large cystic cavity. In addition, there are numerous inflammatory cells in the epithelial space, such as plasma cells and lymphocytes [5, 6]. It’s worth noting that we found out some oncocytic changes in accidental areas of the tumour, just as Hsieh and colleagues reported in 2020 [3, 5], which they named the oncocytic SP relative to the classic SP. Furthermore, the mucosal surface of most cases has many papillary projections supported by fibrovascular connective tissue core, while a few cases only formed numerous micropapillae without a central fibrovascular core [32, 44].

Immunohistochemistry showed that CK7 and CK8 were strongly expressed in columnar luminal cells, and p63 was strongly positive in basal cells, but was negative in luminal cells from Table 2 and reported literatures, and some cases were positive for S-100 and GFAP, which indicate the convoluted ductal structures of SP include two cell types at least [5, 7]. Additionly, in Heieh’s study [3, 5], SOX10 was diffusely and strongly positive in the proliferative ductal cells of classic SP but was completely negative in the oncocytic SP, which suggests the latter may have distinctive cell origin and pathogenesis.

The Histogenesis of SP is still not fully understood [5, 7, 44]. There are several viewpoints as follows. Freedman, Lumerman, and Anuradha et al. proposed that it may originate from the excretory tube cell, which is supposed to be a primitive precursor cell capable of multi-directional differentiation [42, 43]. According to Abrams and Finck, the lesion was of pleuripotential myoepithelial origin because the tumour cells revealed the immunoreactivity for SMA [4]. Moreover, Asahina and others suggested that the lesion derived from the intercalated duct cell due to the presence of the tumour cells coexpressing cytokeratin, vimentin, and desmin [22]. Conversely, Eversole and several authors considered SP as the result of focal hyperplasia after salivary duct obstruction rather than a true neoplasm. Recently, the genetic alteration of SP has been discovered in the reported cases of Hsieh [5] and Nakaguro [44], that is, most SPs have *BRAF V600E* mutations and one case has *HRAS* Q61R mutations, but except oncocytic SP. Notably, syringocystadenoma papilliferum of the skin, histologically analogous to SP, also exists *BRAF* and *HRAS* mutations, which suggests SP may be considered to be a salivary counterpart of syringocystadenoma papilliferum of the skin [3, 5, 44]. Furthermore, in our limited series of SP, BRAF V600E immunoexpression presented in both the proliferative ductal and squamous tumour elements, which confirmed by molecular analysis, similar to the results of Hsieh and Nakaguro [3, 5, 44], suggested the neoplastic nature of both components, and the transition of ductal epithelium to squamous epithelium seen in SP indicated this tumour may originate in the excretory ducts. However, the ductal cells shows SOX10 positivity, suggesting classic SP may be derived from intercalated duct or ductal progenitor cells [3, 5]. Therefore, given the complex histopathological, immunohistochemical and molecular features of SP, its tumor cells may be derived from several components [3, 5, 6, 44]. Despite the cells of origin are not entirely clear at present, BRAF analysis and SOX10 immunostaining can be useful to make a definite diagnosis.

Although SP has been proposed as a distinct entity, it also needs to be differentiated from the following neoplasms. First of all, papillary squamous cell carcinoma, a papillary subtype of squamous cell carcinoma similar to the histopathological characteristics of SP, is characterized by an exophytic and papillary growth pattern. However, there is no glandular component with mucous cells in the lesion, and the squamous cell papilloma is mainly composed of the squamous epithelium, which manifests highly differentiated squamous cell carcinoma with keratinized beads structure, without downward extension of SP. Inverted ductal papilloma is another candidate for differential diagnosis, mainly composed of hyperplastic squamous epithelium under the mucosa that protrudes into connective tissue and connects with duct, but unlike SP with characteristic papillary surface configuration. Another antidiastole is highly differentiated mucoepidermoid carcinoma, which is rich in mucous cells, often forming a glandular cavity, and sometimes hyperplastic mucous cells form the papillary structure resembling that of SP, while mucoepidermoid carcinoma usually composed of epidermal-like cells, intermediate cells, mucus cells and other cell components [14]. Wathin tumour shares papillary adenoid structure with characteristic double layer of epithelium lining the glandular cavity liking SP should also be considered, but the interstitial of it is a lymphoid component associated with lymphoid follicle formation [15]. Also papillary cystadenocarcinoma is a rare malignant tumour characterized by predominantly cystic growth and cell types that comprise the lining epithelium of cysts include most often cuboidal and columnar cells, which though resemble SP, neither exhibits squamous elements.

The current treatment of SP is conservative surgical resection [6, 45]. In addition, Trans-oral robotic surgery (TORS) is a novel technique for head and neck surgery in some centers around the world [46]. Atarbashi-Moghadam proposed the first successful removal of SP tumours by TORS [16]. The use of TORS provides better control of surgical procedures and reduced morbidity compared to traditional oral surgical procedures.

Although there have been a few reports of recurrence and malignant transformation, in which SP has reported that it can transformed into epithelial-muscle epithelial carcinoma, squamous cell carcinoma, and mucoepidermoid carcinoma, but malignant transformation is rare and not entirely convincing [6, 16, 32, 47]. As the results of the statistics show that the prognosis of this lesion is very good, we believe that SP does not have malignant potential [14, 44, 48].

## Conclusions

Summarily, SP is a rare, benign and exophytic tumour of salivary gland neoplasm that commonly occurs in the hard palate in middle aged males with a painless and slow growing lesion. Characteristic of this tumour is its exophytic growth pattern, with multiple papillary surface fronds and deeper ductlike structures, which may be continuous with the surface epithelial component. In this study, we added another five cases of SP to the literature and discussed the clinicopathologic features of the 94 described cases of this unusual neoplasm. Although to identify the cell of origin of SP is difficult, we conclude that SP is a neoplastic lesion by immunophenotypic feature and molecular analysis, and virtually has no potentially malignant features mostly with good prognosis, which should be distinguished from other malignant tumours and avoided resultant overtreatment.

## Data Availability

The data used and/or analyzed during the current study are available from the corresponding author on reasonable request.

## Abbreviations

SP: Sialadenoma papilliferum
WHO: World Health Organization
TORS: Trans-oral robotic surgery
